# Both *OsRecQ1* and *OsRDR1* Are Required for the Production of Small RNA in Response to DNA-Damage in Rice

**DOI:** 10.1371/journal.pone.0055252

**Published:** 2013-01-30

**Authors:** Hui Chen, Kappei Kobayashi, Akio Miyao, Hirohiko Hirochika, Naoto Yamaoka, Masamichi Nishiguchi

**Affiliations:** 1 Faculty of Agriculture, Ehime University, Matsuyama, Ehime, Japan; 2 National Institute of Agrobiological Sciences, Tsukuba, Ibaraki, Japan; Wayne State University, United States of America

## Abstract

Small RNA-mediated gene silencing pathways play important roles in the regulation of development, genome stability and various stress responses in many eukaryotes. Recently, a new type of small interfering RNAs (qiRNAs) approximately 20–21 nucleotides long in *Neurospora crassa* have been shown to mediate gene silencing in the DNA damage response (DDR) pathway. However, the mechanism for RNA silencing in the DDR pathway is largely unknown in plants. Here, we report that a class of small RNAs (qiRNAs) derived from rDNA was markedly induced after treatment by DNA-damaging agents [ethyl methanesulphonate (EMS and UV-C)], and that aberrant RNAs (aRNAs) as precursors were also highly induced after the DNA damage treatment in rice. However, these RNAs were completely abolished in *OsRecQ1* (RecQ DNA helicase homologue) and *OsRDR1* (RNA-dependent RNA polymerase homologue) mutant lines where either gene was disrupted by the insertion of rice retrotransposon *Tos17* after the same treatment. DNA damage resulted in a more significant increase in cell death and a more severe inhibition of root growth in both mutant lines than in the WT. Together, these results strongly suggest that both *OsRecQ1* and *OsRDR1* play a pivotal role in the aRNA and qiRNA biogenesis required for the DDR and repair pathway in rice, and it may be a novel mechanism of regulation to the DDR through the production of qiRNA in plants.

## Introduction

Chromosomal DNA damage in most organisms is caused by two major sources from exogenous factors such as ultraviolet light (UV), ionizing radiation and chemical exposure [1], [2], as well as through endogenous cellular processes such as cellular metabolism and replication errors [3], [4]. Failure to repair DNA damage can lead to blockages of DNA transcription and replication, mutagenesis and cell death [5], [6]. The mechanism of DNA damage response (DDR) and repair is essential for the maintenance of genomic integrity and survival for all organisms [7]. A variety of DNA repair pathways have been developed to fix the different kinds of DNA damage in eukaryotic cells, which mainly repair direct reversal of damage (DR), single-strand breaks (SSBs) and double-strand breaks (DSBs) [4], [5]. The mechanisms of non-homologous end-joining (NHEJ) and homologous recombination (HR) are involved in the DSB repair pathway [8]. Many proteins acting as sensors, transducers or effectors are required for cell cycle checkpoint regulation, DNA repair and apoptosis in different DDR pathways [9]. Previous studies suggest that the RecQ family of DNA helicase is involved in the DNA repair pathway [10], [11]. The anthrax toxin receptor (ATR) and ataxia telangiectasia mutated (ATM) protein kinases have known to be involved in a wide variety of responses to DNA damage in plants [12], both ATM and ATR play central roles in the cellular response to DSBs by regulating DNA repair, cell-cycle arrest and apoptosis [13], and suppressor of gamma response 1 (SOG1) participates in pathways governed by both ATR and ATM sensor kinases in plants [9]. Currently, a novel protein in mammals, RHINO (Rad9, Rad1, Hus1 interacting nuclear orphan) is shown to be required for ATR (ataxia telangiectasia and the Rad3-related) signaling and cell cycle checkpoint activation in the DDR pathway [14], and Wolf-Hirschhorn syndrome candidate 1 (WHSC1) gene in human cells recruited to sites of DNA damage in the DDR [15].

RNA silencing is a manner of gene regulation by degrading sequence-specific RNA, which is conserved among eukaryotes including fungi, animals and plants [16], [17]. A number of genes have been implicated in the diverse RNA silencing pathway in multiple organisms [18], [19], [20]. QDE-1 (RNA-dependent RNA polymerase, RDR homologue) and QDE-3 (RecQ DNA helicase homologue) in the filamentous fungus *Neurospora crassa* have been shown to be involved in the generation of double-stranded RNA (dsRNA) induced RNA silencing [21], [22]. In *Arabidopsis*, some homologues of RDRs (*AtRDR1*, *AtRDR2* and *AtRDR6*) have been shown to be responsible for RNA silencing or the antiviral pathway [23], [24], [25]. In rice, previous studies suggest that *OsRecQ1*, a *QDE-3* homologue, is thought to participated in the process of allowing inverted repeat (IR) DNA to be transcribed into dsRNA that can trigger RNA silencing [26]. *OsRDR1* has been reported to be involved in virus mediated RNA silencing [27], while rice *chromomethyltransferase 3* (*OsCMT3*) has been anticipated to be involved in the epigenetic process in affecting genome activity during abiotic stress [28]. *Suppressor of gene silencing 3* (*SGS3*) in *Arabidopsis* has been shown to be required for the biogenesis of *trans*-acting small interfering RNAs (ta-siRNAs) [24]. There are at least two copies of *SGS3* [*OsSGS3a* (AK064995) and *OsSGS3b* (AK100699)] in rice, and a recent finding showed that rice (*OsSGS3a*) interacted with a silencing suppressor, *Rice stripe virus* (RSV) p2 protein, which has been demonstrated to be be targets of *TAS3*-derived ta-siRNAs, was up-regulated in RSV-infected rice [29].

Small RNA (sRNA), including small interfering RNA (siRNA) and microRNA (miRNA), plays an important role in the RNA silencing pathway during diverse biological processes in plants and animals [30], [31]. Later studies have shown that siRNA and miRNA are mobile signals that control gene regulation in the RNA silencing pathway [32], [33]. More recently, small RNA-mediated gene silencing as a new layer has been shown to modulate protein activity in the DDR pathways in *Neurospora* and animals [34], [35], [36], but the mechanism for RNA silencing in the DDR pathway remains largely unknown in plants. In this paper, we present the role of *OsRecQ1* and *OsRDR1* in the small RNA regulating DDR in rice and propose a novel mechanism of gene regulation to the DDR through small RNA biogenesis in plants.

## Results

### qiRNAs Induced by DNA-damaging Agents in Rice

**Figure 1:**
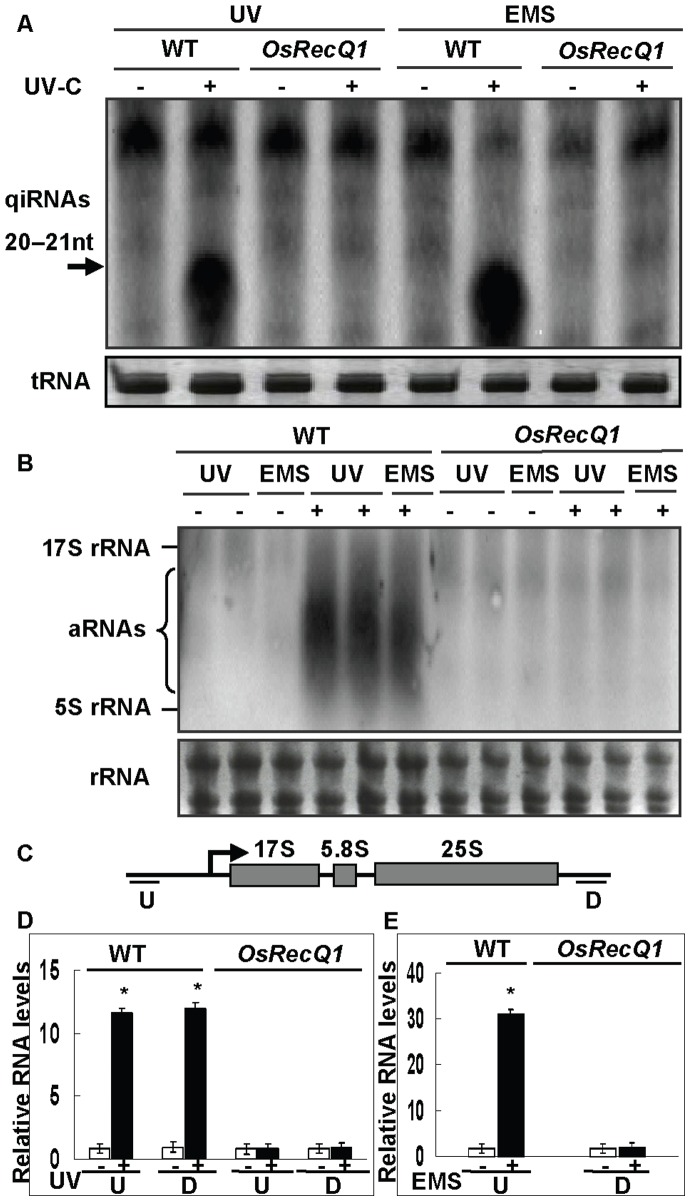
The detection of aRNAs and qiRNAs in WT and the *OsRecQ1* mutant line (ND8004) after the DNA damaging agent UV or EMS treatment by northern blot and RT-qPCR analysis. (A) The results show a significant induction of qiRNAs about 20–21 nucleotides (nt) in length after UV or EMS treatments in WT, but a complete abolishment in the *OsRecQ1* mutant line. An RNA probe derived from the sense 25S rDNA region was used. The arrow denotes the qiRNAs. The bottom panel of tRNAs shows equal loading control. (B) The results show a marked induction of 25S rDNA specific transcripts after UV or EMS treatments in WT, but a complete loss in the *OsRecQ1* mutant line (ND8004). An RNA probe derived from sense 25S rDNA region was used. The bottom panel of rRNAs shows equal loading control. (C) A schematic diagram shows the upstream (U) and downstream (D) primers from rice rDNA regions for RT-qPCR analysis. The transcriptional start site is shown with an arrow. (D) RT-qPCR results indicating an abolishment of aRNAs from the rDNA locus induced by UV treatment in the *OsRecQ1* mutant lines (ND8004). The expressing level of rice ubiquitin gene was used as the internal control. Two independent experiments are shown. Data are the mean ± SE (n = 3), *P<0.001. RT-qPCR analysis showing a loss of aRNAs from the rDNA locus induced by EMS treatment in the *OsRecQ1* mutant lines (ND8004).

A previous study has shown that a new class of small interfering RNAs (qiRNAs) in *Neurospora crassa* is involved in regulation of gene silencing in the DDR pathway [36]. It is worthwhile examining whether there is a similar mechanism of qiRNA regulation to the DDR in plants. Here, rice leaves and calli were treated by the DNA-damaging agents EMS or UV-C, respectively. Northern blot analysis with an RNA probe specific for the antisense 25S rDNA region showed that a class of small RNAs (qiRNAs) about 20–21 nucleotides (nt) in length was significantly induced after UV or EMS treatment, but it was at an undetectable level under normal conditions in WT (Figure 1A), and a similar result was also obtained using an RNA probe specific for the sense 25S rDNA region (data no shown), suggesting that these small RNA are double stranded. Interestingly, qiRNA accumulation was completely abolished in the *OsRecQ1* mutant line (ND8004) (Figure 1A). These results suggest that *OsRecQ1* is required for qiRNA biogenesis in the DDR pathway.

### aRNAs Required for qiRNA Biogenesis as Precursors in Rice

It is generally accepted that the biogenesis of qiRNAs requires aberrant RNAs (aRNAs) as precursors [36]. To examine this possibility, the relationship between qiRNAs and aRNAs was investigated in rice by northern blot analyses and quantitative reverse transcription PCR (RT-qPCR). The results from the northern blot analyses using an RNA probe specific for the antisense 25S rDNA region show that aRNAs derived from rDNA specific transcripts with a few hundred nucleotides (nt) to 2 kilobases (kb) were markedly induced after UV or EMS treatments in WT, but were completely abolished in the *OsRecQ1* mutant line (Figure 1B) and a similar result was also obtained using an RNA probe specific for the sense 25S rDNA region (data no shown) suggesting that these aRNAs derived from both strands of DNA for 25S rRNA. RT-qPCR analyses showed that aRNAs originated from both upstream (U) and downstream (D) regions of the transcribed 25S rDNA locus were highly induced after UV or EMS treatment, but were completely abolished in the *OsRecQ1* mutant lines (Figure 1D and E). These results indicate that aRNAs transcribed from the rDNA locus as precursors are required for qiRNA biogenesis in the DDR in rice and that the RecQ DNA helicase, *OsRecQ1*, is required for aRNA biogenesis in the DDR pathways.

### *RNA-dependent RNA Polymerase 1* (*RDR1*) Required for aRNA and qiRNA Biogenesis in Rice

**Figure 2:**
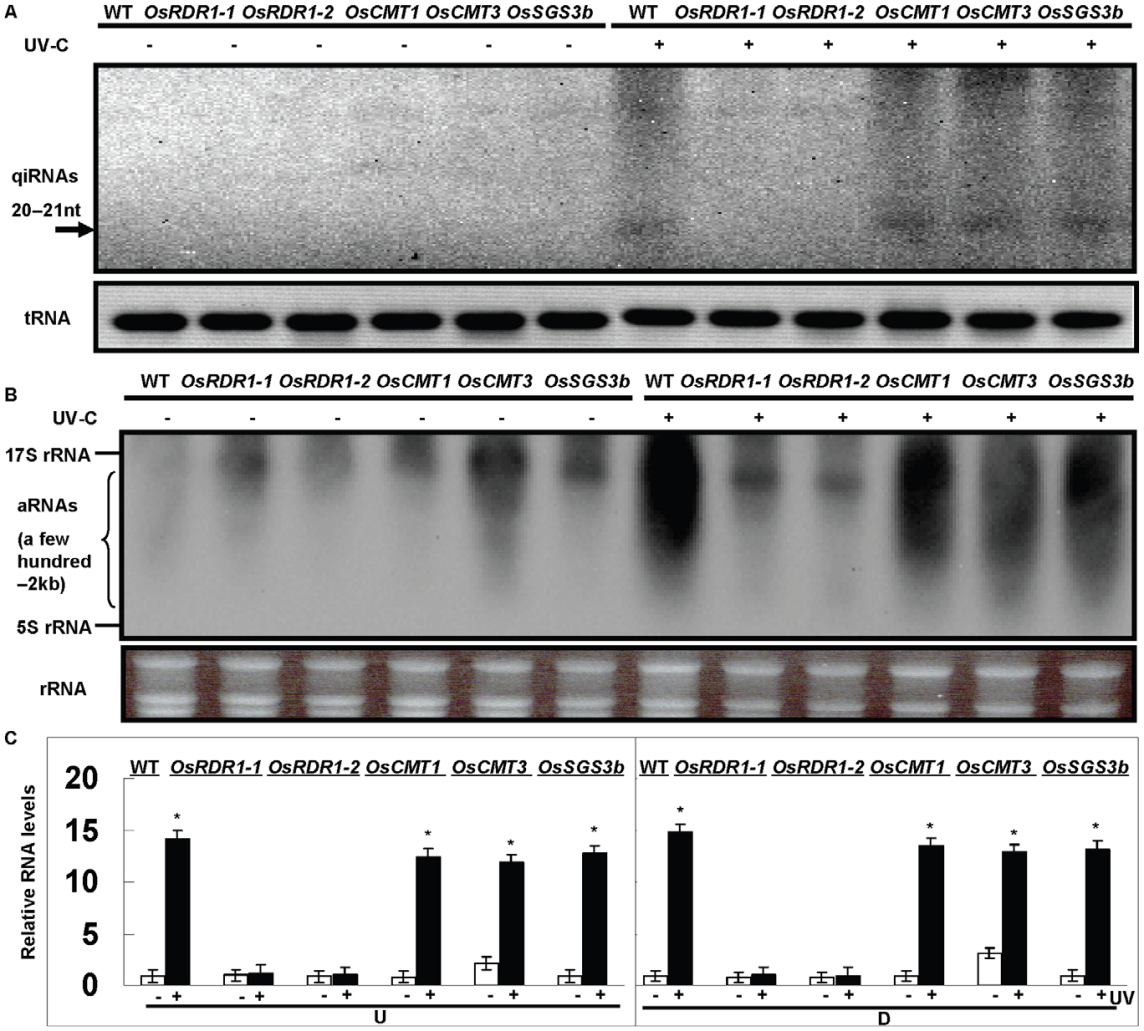
The detection of aRNAs and qiRNAs in WT and other mutant lines after UV treatment by Northern blot and RT-qPCR analysis. (A) The results show an obvious induction of qiRNAs after UV or EMS treatments in WT and some of the mutant lines, but a complete abolishment in both *OsRDR1* mutant lines. An RNA probe derived from the sense 25S rDNA region was used. The arrow denotes the qiRNAs. The bottom panel of tRNAs shows equal loading control. *OsRDR1-1*(ND2001), *OsRDR1-2*(ND2059), *OsCMT1* (NE7010), *OsCMT3* (NC4949) and *OsSGS3b* (NE5050) mutant lines were used (see experimental procedures). (B) The results show a high induction of rDNA specific transcripts after UV treatment in WT and some of mutant lines, but an obvious loss in both *OsRDR1* mutant lines (ND2001 and ND2059). An RNA probe derived from sense 25S rDNA region was used. The bottom panel of rRNAs shows equal loading control. *OsRDR1-1*(ND2001), *OsRDR1-2*(ND2059), *OsCMT1* (NE7010), *OsCMT3* (NC4949) and *OsSGS3b* (NE5050) mutant lines were used (see experimental procedures). (C) The results show an abolishment of aRNAs from the rDNA locus induced by UV treatment in both *OsRDR1* mutant lines (ND2001 and ND2059). The expressing level of rice ubiquitin gene was used as the internal control. Data are the mean ± SE (n = 3), *P<0.001. *OsRDR1-1*(ND2001), *OsRDR1-2*(ND2059), *OsCMT1* (NE7010), *OsCMT3* (NC4949) and *OsSGS3b* (NE5050) mutant lines were used (see experimental procedures).

RDRs are an essential component of RNA silencing and can specifically recognize aRNAs, convert them to double-stranded RNA (dsRNA) [37], [38]. Some other related genes such as *OsCMT3* [28]and *OsSGS3* [29] may participate in this biological processing of DDR. To examine this possibility in the DDR in rice, the accumulation of qiRNAs and aRNAs was investigated by northern blot analyses and RT-qPCR detection in the *OsRDR1* as well as other mutant lines including *OsCMT1*, *OsCMT3* and *OsSGS3b* [24], [28], [29]. The results from the northern blot analyses with a RNA probe specific for the antisense 25S rDNA region show that qiRNAs and aRNAs were obviously induced after UV treatment in WT, *OsCMT1*, *OsCMT3* and *OsSGS3b* (Figure 2A and B). Notably, qiRNAs and aRNAs were completely abolished in both *OsRDR1* mutant lines (ND2001 and ND2059) [25] but not in other mutant lines (Figure 2A and B), and a similar result of qiRNAs and aRNAs was also obtained using a RNA probe specific for the sense 25S rDNA region (data no shown. Furthermore, RT-qPCR analysis also showed that aRNAs derived from both the upstream (U) and downstream (D) regions of the rDNA locus were significantly induced after UV treatment in WT, *OsCMT1*, *OsCMT3* and *OsSGS3b* (Figure 2C), but not in the *OsRDR1* mutant lines, indicating that *OsRDR1* is indispensable for qiRNA and aRNA biogenesis in the DDR pathway in rice.

### DDR Induced by DNA-damaging Agents in Rice

**Figure 3:**
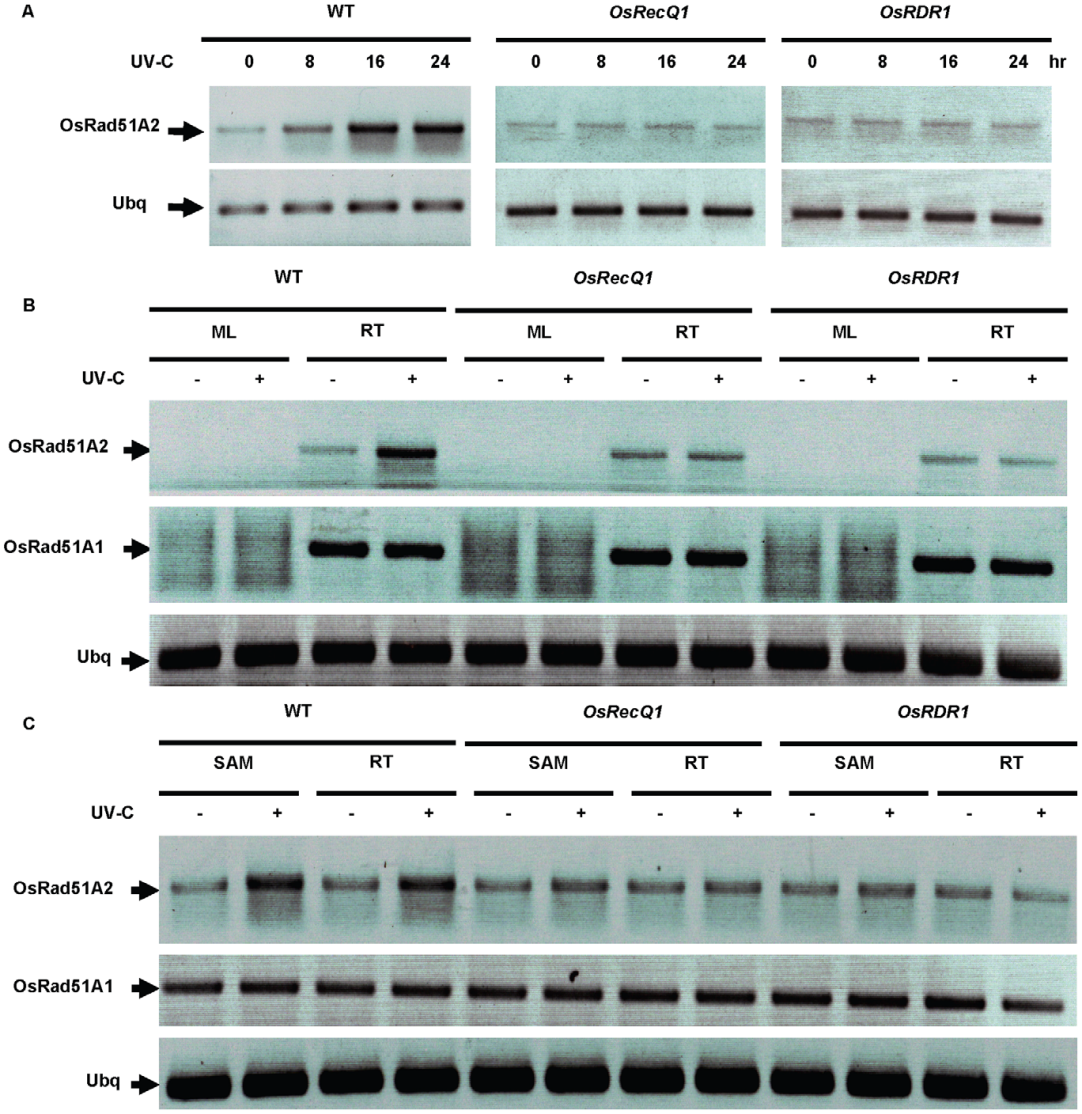
The expression of DDR genes induced by UV-C treatments in rice. (A) An increasing expression of *OsRad51A2* induced by UV-C irradiation in the root tip tissues (RT) of WT, but not in the *OsRecQ1* (ND0059) and *OsRDR1* (ND2001) mutant lines. Levels of the rice ubiquitin gene were used as the internal control. (B) A strongly detection of *OsRad51A2* in the RT after UV-C treatment in WT, but not in the *OsRecQ1* (ND0059) and *OsRDR1* (ND2001) mutant lines, and the transcription of *OsRad51A1* was not changed after UV-C treatment in the RT and the mature leaf (ML). Levels of the rice ubiquitin gene were used as the internal control. (C) A highly expression of *OsRad51A2* in the shoot apical meristem (SAM) and the RT after UV-C treatment in WT, but not but not in the *OsRecQ1* (ND0059) and *OsRDR1* (ND2001) mutant lines, and the levels of *OsRad51A1* transcription was not different before and after UV-C treatment in the SAM and the RT. Levels of the rice ubiquitin gene were used as the internal control.

To investigate the DDR in the *OsRecQ1* and *OsRDR1* mutant lines, two-week-old seedlings were treated by UV-C irradiation for 0, 8, 16, 24 h. In *Arabidopsis*, *Rad51A* gene family is found to be involved in the DDR [4], [5]. After the treatments, the expression of *OsRad51A1* and *OsRad51A2* were checked by RT-PCR analysis, and the results show that the transcription of *OsRad51A2* was strongly induced by UV-C irradiation in the root tip (RT) of WT, but not in the *OsRecQ1* and *OsRDR1* mutant lines (Figure 3A), and *OsRad51A2* was also strongly detected in the shoot apical meristem (SAM) after UV-C treatment, but not in the mature leaf (ML) possibly because of no proliferating stage (Figure 3B and C). However, the transcription of *OsRad51A1* was not changed in the RT and SAM after UV-C treatment (Figure 3B and C). These results suggest that *OsRad51A2*, not *OsRad51A1,* may participate in the DDR pathway induced by UV-C.

**Figure 4:**
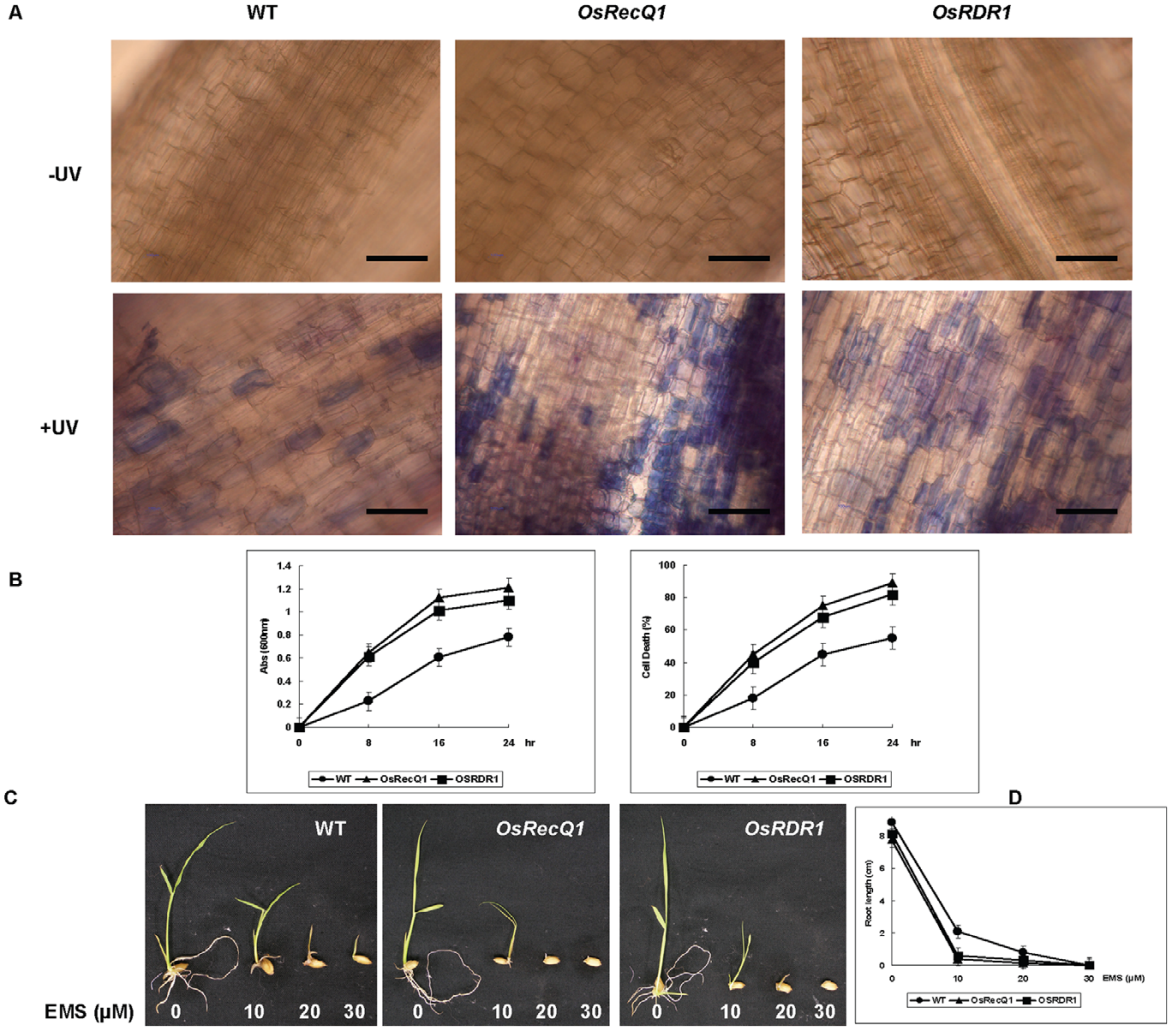
Measurement sensitivity to UV-C and EMS treatments in rice. (A) The results showing an increased number of dead cells stained by Evan’s blue under the microscope in the *OsRecQ1* (ND0059) and *OsRDR1* (ND2001) mutant lines. (B) UV-C irradiation resulted in a more significant increase in cell death in the root tips of *OsRecQ1* (ND0059) and *OsRDR1* (ND2001) mutant lines than in the WT. Data are the mean ± SE (n = 3). (C) Sensitivity of the *OsRecQ1* (ND0059) and *OsRDR1* (ND2001) mutant lines to EMS. The EMS dosage is shown (0, 10, 20, 30 µM). Twenty seeds were tested in each assay. Three independent experiments were carried out. (D) Root growth of seedlings in the *OsRecQ1* (ND0059) and *OsRDR1* (ND2001) mutant lines with a severe suppression after EMS treatments compared to the WT. Data are an average of triplicate assays and bars indicate the mean ± SE (n = 3).

To further examine the DDR, the root tissues after UV-C treatment were checked for cell death by Evans blue staining. The results showed that more dead cells were stained blue under the microscope (Figure 4A), suggesting the higher frequency of cell death in the both *OsREcQ1* and *OsRDR1* mutant lines than that in the WT.

Meantime, the suspension cell assay was also performed by Evans blue staining for monitoring cell death estimation by spectrophotometry or by microscopic observation [39], and both results indicate that UV irradiation resulted in a more significant increase in cell death in the *OsRecQ1* and *OsRDR1* mutant lines than in the WT (Figure 4B). Moreover, the root growth assay of seedlings [40] was carried out by liquid cultures containing 0, 10, 20, 30 µM EMS, and the results show that the root growth of the *OsRecQ1* and *OsRDR1* mutant seedlings was more severely suppressed compared to the WT (Figure 4C and D). These results indicate that *OsRecQ1* and *OsRDR1* play a key role in the DNA repair pathway.

## Discussion

The dynamic state of DNA metabolism acts as replication, recombination and repair for tolerating and repairing numerous types of damage in all living organisms [5]. Although failure to repair DNA damage can lead to serious diseases in humans and animals, this situation is not a particular case in most higher plants. However, the mechanism of DNA metabolism plays a significant role in cell metabolic activity, normal growth and development in reproductive tissues of higher plants such as meristematic tissues [4]. *Rad51-like* genes were previously shown to be involved in HR and related repair pathways through mediating strand invasion and exchange between homologous DNA molecules [4], [5]. The transcription of *OsRadA* is thought to be related to the level of cell proliferation in the meristematic tissues [41] and for meiotic homologous recombination and the repair of DSBs [42], [43], [44]. During the study of *OsRecQ1* and *OsRDR1* functions in the DDR, we observed that the expression of *OsRad51A2* was significantly induced by UV-C irradiation in the RT and SAM of WT, but not in the *OsRecQ1* and *OsRDR1* mutant lines (Figure 3A). These results suggest that the transcription of *OsRad51A2* is particularly relevant to the level of cell proliferation in the replicating tissues, but that of *OsRad51A1* is not (Figure 3B and C), suggesting that *OsRad51A1* may has a different role from that of *OsRad51A2* in this DDR pathway. It can be thought at least that *OsRecQ1* and *OsRDR1* are in the upstream to *OsRAD51A2*. Since these two genes are involved in qiRNA biogenesis, qiRNA might affect the transcription of *OsRDA51A2*. It remains to elucidate how qiRNA (or other yet unknown *OsRDR1*and *OsRecQ1* involved small RNA) are directly/indirectly involved in the transcription of *OsRAD51A2*, and that *OsRecQ1* and *OsRDR1* may play an important role in the processing of cell proliferation in the DDR pathway. It would be interesting to investigate whether this is general for other genes related to the DDR such as BRCA1 (Breast Cancer 1), MRE11 (Meiotic Recombination 11) or RECQ4 (Recombination Q4) in rice.

**Figure 5:**
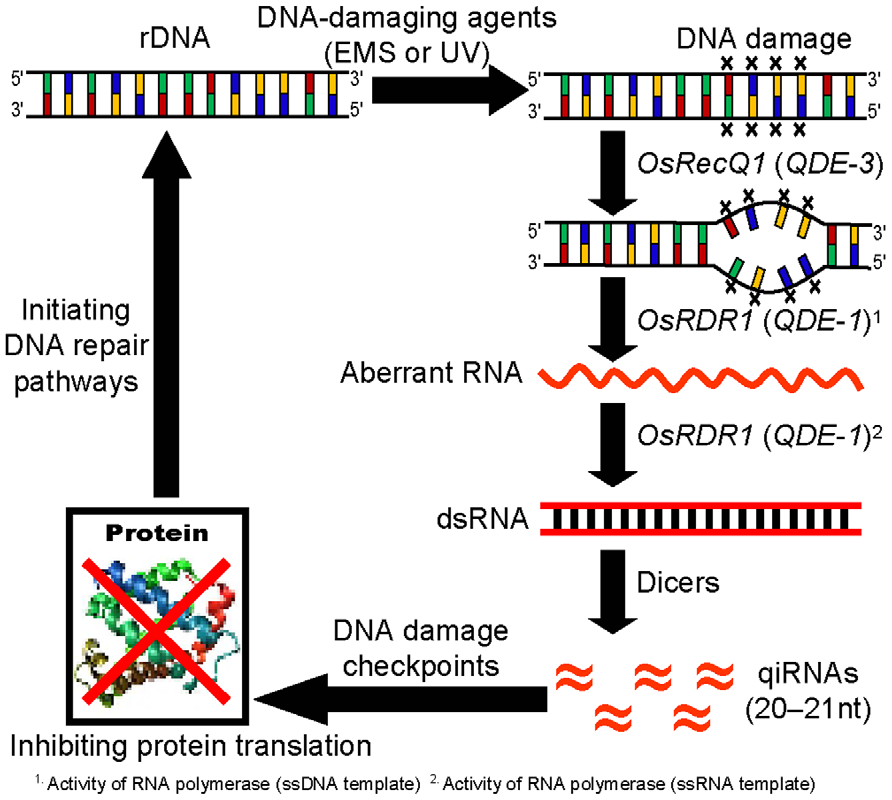
A proposed model for the RNA silencing pathway in the DNA-damage response. *OsRecQ1* (QDE-3 in *Neurospora crassa*) and *OsRDR1* (QDE-1 in *Neurospora crassa*) are required for both aRNA and qiRNA biogeneses, respectively, after DNA-damaging agent (EMS or UV) treatment. qiRNAs may participate in a novel mechanism of regulation to the DDR in plants.

Although the mechanisms of DNA damage and repair have been clearly established in bacteria, yeast, and mammals, it is worthwhile determining whether these mechanisms exist in higher plants [4]. As a new class of small RNA, qiRNA, has recently been shown to be involved in regulation of gene silencing in the DDR pathway in *Neurospora crassa* [36]. The present research focuses on a novel aspect of small RNA-mediated gene silencing in the DDR pathways in rice. The results suggest that the production of qiRNA may be a novel mechanism for the DDR in plants, which is similar to the mechanism of RNAi in the DDR pathway in *Neurospora crassa*. In Figure 5, a proposed model for the RNA silencing pathway in the DDR is shown. After DNA damage, cells activate the DNA repair pathway that decides the cell’s fate either to repair damage or to undergo apoptosis [34], [36]. The DDR provokes cell-cycle progression to regulate protein levels through the small RNA-mediated gene silencing pathway, which responds to DNA damage checkpoints [36], [45]. Our results show that both *OsRecQ1* and *OsRDR1* are required for aRNA and qiRNA biogenesis after DNA-damaging agent (EMS or UV) treatments, and aRNAs are required for qiRNA biogenesis as precursors. In our experiments not only qiRNA but also aRNA were found to be double-stranded because either sense or antisense RNA probe could detect their RNA bands in the northern blots. aRNA is thought to be single-stranded [46], [47]. However, it is not the case in this study. It may possible that in DDR both sense and antisense DNA strands at the same rDNA locus could be transcribed to produce aberrant RNAs. DNA helicase and RDR may participate in this step because QDE1 showed RNA/DNA dependent RNA polymerase activity [36] and RDR6 in *Arabidopsis* showed polymerase activity on ssRNA as well as ssDNA in *in vitro* polymerase activity assay [37]. It remains to be determined whether both sense and antisense 25S rDNA regions were transcribed as a unit length or not.

More recently, a kind of small RNAs (DSB-induced small RNAs, diRNAs) has been reported to be involved in the DSB repair pathway. diRNAs are generated from the sequences in the vicinity of DSB sites in *Arabidopsis* and in human cells [48]. In our current findings, qiRNAs are derived from rDNA repeats, which contribute to DDR by inhibiting rRNA biogenesis and regulating protein translation levels [36]. Upon exposure to DNA damaging agents, rDNA-specific small RNAs are induced that mediate DSB repair on damaged repetitive rDNAs. Therefore, qiRNAs are different kind of small RNAs in the DDR pathway from diRNAs in the DSBs repair pathway.

In summary, we demonstrated that both *OsRecQ1* and *OsRDR1* are required for aRNA and qiRNA biogenesis in the DDR pathway, qiRNAs derived from rDNAs repeats are important for efficient DDR. It will be very exciting to have further studies on dissecting the mechanisms by which the production of small RNAs participate in the DDR pathways in plants.

## Materials and Methods

### Plant Materials and Growth Conditions

The WT (*Oryza sativa* L. cv. Nipponbare) and its knockout mutant lines were used in this study. *OsRecQ1* (ND8004 and ND0059) and *OsRDR1* (ND2001 and ND2059) mutant lines were reported previously [26], [27]. *OsSGS3b* (NE5050), *OsCMT1* (NE7010) and *OsCMT3* (NC4949) mutant lines were used in this study. RNA silencing induction by particle bombardment was defective in these three mutants (unpublished data). The seeds of homozygous mutant lines were used to produce calli, and the plants and its calli were grown in proper conditions as previously described [26].

### Plant Sensitivity Measurement to UV-C and EMS Treatments

Two-week-old seedlings were irradiated under ultraviolet light (UV-C, 254 nm) using a germicidal lamp (Matsuda) for 0, 8, 16, 24 h as previously described [40]. Total RNA and mRNA were isolated from the rice tissue of ML, SAM or RT after UV-C treatment. RT-PCR analysis was performed as previously described [26]. Levels of the rice ubiquitin gene were used as the internal control. The sequences of primer pairs for RT-PCR for *OsRAD51A1* (AB080262) and *OsRAD51A2* (AB080264) genes were amplified by the following pairs of primers: 5′-GCTCATGCTTCCACAACAAG-3′ (OsRad51-F), 5′-GGCAGAAAACTTACTTCG-3′ (OsRad51A1-R) and 5′-AATTCTGGCTCGTCTAAC-3′ (OsRad51A2-R), respectively.

For the root tissues staining by the Evan’s blue and suspension cell assay, aliquots of RT were removed from treatments and performed by Evan’s blue assay for monitoring cell death estimation by spectrophotometry or by microscopic observation as previously described [39].

For the root growth assay, 20 seeds of rice WT and each mutant line were grown in a Petri dish for liquid cultures containing 0, 10, 20, 30 µM EMS (Sigma-Aldrich) for two weeks with a modified version as previously described [41]. Three independent experiments were carried out.

For the detection of qiRNAs and aRNAs, four leaf segments (about 6 cm) of seedlings at the two-week-old stage or one-month-old calli were used for the treatments by irradiation under UV-C light for 24 h or by liquid cultures containing 0.4% EMS (Sigma-Aldrich) for 48 h. After the treatments, northern blot and RT-qPCR analyses were performed as described below.

### RNA Gel Blot Analyses

Total RNA was extracted from rice leaves and calli after DNA damage treatments as previously described [26], [27]. Low and high molecular weight RNAs were used to detect qiRNAs and aRNAs with 25 and 40 µg of total RNA, respectively. Sense or antisense rRNA probes were prepared by in-vitro transcription derived from 25S rDNA regions (2035 bp fragment from 3 to 2037 region in AK119809) using a DIG RNA labeling (SP6/T7) kit (Roche) following the manufacturer’s instructions. For small RNA detection, the RNA probe was hydrolyzed to an average size of 50 nt as described [49], [50]. Prehybridization and hybridization were performed for qiRNA and aRNA detection at 42°C or 65°C, respectively. After hybridization, the membrane was washed three times with 0.1× SSC and 0.1% SDS buffer for 30 min at 50°C or 68°C and detected by a DIG detection kit (Roche) following the manufacturer’s instructions.

### RT-qPCR Analyses

Quantitative real-time PCR (RT-qPCR) was performed with a Light Cycler II system (Roche) using a previously described protocol [26], [27]. In brief, total RNA was isolated using an RNeasy plant mini kit (QIAGEN) and treated with RNase-free DNase I (Roche), and Poly (A)^+^ mRNA was purified by an Oligotex-dT30 mRNA purification kit (TaKaRa). Reverse transcription using random hexamers was carried out with equal amounts of mRNA (100 ng) by an AMV reverse transcriptase XL kit (TaKaRa). Synthesized cDNAs were 10-fold diluted and used for PCR by the incorporation of the fluorescent DNA dye SYBR green using the QuantiTect™ SYBR® Green PCR kit (Qiagen). Gene-specific primers were derived from the upstream (forward, 5′-AGTCCCCAGGCCTCTCTAAG-3′ and reverse, 5′-GTCCCGTCCTTGGAGTCTG-3′ 103 bp fragment from 61 to 163 region in X58275) or downstream (forward, 5′-CGATGTCGGCTCTTCCTATC-3′ and reverse, 5′-AACCTGTCTCACGACGGTC-3′ 105 bp fragment from 7892 to 7997 region in AP008245) sequence of the 25S rDNA region. Each reaction was performed in duplicate. Levels of the rice ubiquitin gene were used as the internal control [26].
